# Association of medication clusters and subsequent labor market marginalization in refugee and Swedish-born young adults with common mental disorders in Sweden

**DOI:** 10.1007/s00787-023-02214-8

**Published:** 2023-04-28

**Authors:** J. Chen, E. Mittendorfer-Rutz, H. Taipale, S. Rahman, T. Niederkrotenthaler, P. Klimek

**Affiliations:** 1 Section for Science of Complex Systems, CeDAS, Medical University of Vienna, Vienna, Austria; 2 Complexity Science Hub Vienna, Vienna, Austria; 3 Division of Insurance Medicine, Department of Clinical Neuroscience, Karolinska Institutet, 171 77 Stockholm, Sweden; 4 Niuvanniemi Hospital, Kuopio, Finland; 5 University of Eastern Finland, School of Pharmacy, Kuopio, Finland; 6 Unit Suicide Research and Mental Health Promotion, Department of Social and Preventive Medicine, Centre for Public Health, Medical University of Vienna, Vienna, Austria; 7 Wiener Werkstätte for Suicide Research, Vienna, Austria

**Keywords:** Psychotropic medication, Mental disorder, Refugee, Young adult, Clustering

## Abstract

Little is known about the association between common mental disorders (CMD) and labor market integration among refugee and Swedish-born young adults. Socially disadvantaged patients such as refugees are more likely to discontinue their medication use prematurely. This study aimed to identify clusters of individuals with similar psychotropic medication patterns; and examine the relationship between cluster membership with labor market marginalization (LMM) in refugee and Swedish-born young adults with CMD. The study uses a longitudinal matched cohort aged 18-24 years with CMD diagnoses from Swedish registers covering 2006–2016. Dispensed psychotropic medications (antidepressants, antipsychotics, anxiolytics, sedative-hypnotics, mood stabilizers) were collected one year before and after CMD diagnosis. Clusters of patients with similar time courses of prescribed dosages were algorithmically identified. The association of cluster membership with subsequent LMM, (long-term sickness absence, SA, disability pension, DP, or long-term unemployment, UE) was assessed using Cox regression. Among 12,472 young adults with CMD, there were 13.9% with SA, 11.9% with DP, and 13.0% with UE during a mean follow-up of 4.1 years (SD 2.3 years). Six clusters of individuals were identified. A cluster with a sustained increase in all medication types yielded the highest hazard ratio (HR [95% CI]) 1.69 [1.34, 2.13] for SA and 2.63 [2.05, 3.38] for DP. The highest HRs of UE give a cluster with a concentrated peak in antidepressants at CMD diagnosis (HR 1.61[1.18, 2.18]). Refugees and Swedish-born showed similar associations between clusters and LMM. To prevent LMM, targeted support and early assessment of CMD treatment are needed for individuals with a sustained increase in psychotropic medication after CMD diagnosis and for refugees in high-risk clusters for UE characterized by a rapid lowering of treatment dosages, which could be an indicator for premature medication discontinuation.

**Supplementary Information:**

The online version contains supplementary material available at 10.1007/s00787-023-02214-8.

## Introduction

Labor market marginalization (LMM) among young people is a growing public health and economic concern in Europe [1]. Different definitions of LMM have been adopted in earlier literature. Unemployment is the most frequently used measurement for LMM, yet the number of individuals excluded from the labor market may be underestimated by only applying this measure [2]. Among other definitions, using long-term sickness absences (SA), disability pension (DP), in addition to long-term unemployment (UE) as indicators of LMM could better account for the burden of LMM due to health conditions [2–4]. In Sweden, the number of young adults being granted DP has been rising in recent years [5], and the unemployment rate is also high among youth in Sweden [1]. Among the risk factors that contribute to these worrying trends, mental disorders may play an important role in the pathways to LMM of young adults [2–4]. Mental disorders are the major disease burden among unemployed youth and youth on welfare benefits [5, 6], and are strongly associated with functional and occupational impairment [7]. In particular, socially disadvantaged youth such as refugees showed increased vulnerability to LMM and worse mental health than their peers [2, 4, 6, 8].

The refugee population is rising globally, with an estimated 42% of forcibly displaced refugees being minors in 2020 [9]. Traumatic pre- and post-migration experiences have been found to correlate with a higher prevalence of common mental disorders (CMD) among refugees, including symptoms of depression, anxiety, and post-traumatic stress [8, 10]. This is particularly the case in refugee youth experiencing an early onset of CMD [10]. CMD in young migrants is a known risk factor for labor market withdrawal [11], loss in disability adjusted life year (DALY) [7], and DP. [2, 4]

Psychotropic drugs, such as antidepressants, antipsychotics, and anxiolytics, are often prescribed to treat CMD. Sedative-hypnotics and mood stabilizers are subclasses of psychotropic drugs used, respectively, for anxiety disorders or sleep, and mood disorders. However, adherence to medication in CMD patients is often suboptimal [12], with socially disadvantaged patients such as refugees being more likely to discontinue their medication use prematurely [13, 14]. It was further reported that young refugees have a lower healthcare utilization for CMD and are less likely to initiate antidepressant treatment compared to the majority population in Sweden [15, 16]. Poor adherence to medication regimens could further increase the severity of CMD [12], and potentially increase the risk of LMM among young refugees*.*

Here, we seek to identify typical patterns of concurrent use of different psychotropic medications over time by algorithmically assigning them to clusters of patients with similar medications over time. To this end, we use a computational clustering algorithm for which it is not necessary to prespecify the expected number of clusters or their structure [17]. Little is known about the relationship of such medication patterns with UE, DP, and SA, and whether the association of different psychotropic medication patterns with LMM differs by refugee status. It is thus important to address these knowledge gaps to fully understand the types and time courses of medications to aid the development of new strategies to prevent LMM in young refugees. In this study, we hypothesize that different clusters of psychotropic medications (antidepressants, antipsychotics, anxiolytics, sedative-hypnotics, and mood stabilizers) show heterogeneous associations with subsequent LMM in refugees compared to Swedish-born youth in Sweden. In particular, we hypothesize that medication use patterns that are consistent with premature discontinuation of medications show a higher risk for LMM than clusters without such signs of discontinuation.

### Aims

The aim of this study was to (i) identify clusters of patients with similar psychotropic medication patterns over a two-year period centered on the time of CMD diagnosis and (ii) examine the relationship between the identified medication clusters and LMM after this period in refugee and Swedish-born young adults with CMD.

## Methods

### Study population

We combined data from Statistics Sweden, the National Board of Health and Welfare, and the Social Insurance Agency for a longitudinal prospective cohort study. The study population included refugee youths, aged 18–24 years at the time of diagnosis with CMD for the first time between January 1st, 2007 and December 31st, 2013, and residing in Sweden from one year before and after CMD diagnosis (exposure period). CMD was defined by the International Classification of Diseases version 10 (ICD-10) code ranges F32–F33 and F40–F43 from inpatient or specialized outpatient care records. Specific CMDs were defined by (ICD10) depressive disorder (F32-F33), anxiety disorder (F40-43 excluding F43.1), and post-traumatic stress disorder (PTSD, F43.1). Each refugee was matched with five Swedish-born youths by age, gender, residence region, year of CMD diagnosis, and type of CMD (depression/PTSD/anxiety disorder). The exposure measure was based on the dispensing of psychotropic medication collected during the exposure period (1 year prior and 1 year after diagnosis of CMD). Individuals with ongoing DP at the end of the exposure period were not included in the study. From the study base of 2921 refugee and 13,911 Swedish-born young adults, 4360 individuals were excluded due to not having been dispensed medications during the exposure period. Thus, 12,472 individuals, consisting of 1454 refugee and 11,018 Swedish-born young adults with CMDs, met the inclusion criteria. Ethics approval was acquired by the Regional Ethical Review Board, Karolinska Institutet, Stockholm (nr 2007/762-31).

### Data sources

Information on socio-demographic factors, unemployment, and social benefits were obtained from the Longitudinal Integration Database for Health Insurance and Labor Market Studies (LISA). The reason for settlement in Sweden was obtained from the longitudinal database for integration studies (STATIV). The country of birth of parents was recorded from the Multi-Generation register. Date and cause of hospital admission, in addition to information on specialized outpatient care, were obtained from The National Patient Register. Death date and cause of death were collected from the Cause of Death Register. Prescribed psychiatric medication, including defined daily doses (DDDs) and dates of dispensing, was established through the Prescribed Drug Register. Micro Data for Analysis of Social Security (MiDAS) held at the Social Insurance Agency recorded the date, grade and duration of SA, as well as the date of DP.

### Refugee status

The study population was grouped into refugee and Swedish-born young adults. Refugee status was defined by the grant of a resident permit in Sweden as a refugee under the Geneva Convention or the grant of a resident permit on “humanitarian grounds,” “in need of protection,” or for “family reunification” reasons [15]. Swedish-born youth were defined as individuals born in Sweden with both parents born in Sweden. Individuals with different foreign backgrounds (e.g., non-refugees that were not born in Sweden) were not included in the analysis.

### Exposure assessment

The exposure of interest was medication cluster membership, which was assigned according to time patterns of medication use within the exposure period. Dispensed prescription medications according to the Anatomical Therapeutic Chemical Classification (ATC) were assessed within the exposure period. The medication categories of interest (ATC codes) were antidepressants (N06A), antipsychotics N05A, excluding lithium), anxiolytics (N05B), sedative-hypnotics (N05C), and mood stabilizers (carbamazepine N03AF01, valproic acid N03AG01, lamotrigine N03AX09, lithium N05AN01). A detailed description of the computation method is provided in the Statistical Analysis section.

### Outcome assessment

The follow-up period for outcomes started one year after the initial CMD diagnosis, the end of the exposure period. LMM was evaluated by SA (> 90 annual net days), DP, or UE (> 180 annual days), followed from one year after CMD diagnosis until the first date of granted SA, DP, UE, December 31st, 2016, emigration, or death, whichever occurred first. UE is based on the annual number of days being registered as unemployed. We utilized the previously used definition of > 180 days to define UE [2], to exclude temporal short periods of unemployment which may not describe the similar difficulty level of finding a job.

### Other covariates

Socio-demographics, previous healthcare utilization (inpatient/specialized outpatient care), and history of diagnoses were assessed at baseline, i.e., at end of the exposure period, including age (continuous, years), sex (female or male), education (0–9 years, 10–12 years, > 12 years), and residence region (big cities, medium-sized cities, small cities/villages). History of comorbid diagnoses during the three years prior to the baseline, including personality disorder (ICD-10 codes F60-69), substance use disorder (ICD-10 codes F10-19, without F17), eating disorder (ICD-10 code F50), attention deficit hyperactivity disorder (ADHD; ICD-10 code F90, ATC: N06BA01, N06BA02, N06BA04, N06BA07 and N06BA09), were established through ICD-10 or ATC codes.

### Statistical analysis

A cluster analysis was used to classify individuals with a similar time-dependent psychotropic drug prescription using the Louvain clustering algorithm (see SI.) [17] Sensitivity analysis on the clustering approach was performed (i) by examining refugee or Swedish-born young adults separately, and ii) by examining individuals with anxiety or depressive disorder separately.

We applied crude and multivariate Cox regression to examine the relation between the cluster variable (medication group) and LMM. The multivariable model was adjusted for refugee status (in those analyses not stratified for refugee status), education, and history of SA and UE (yielding Hazard ratios, HRs and 95% Confidence Intervals, CI). Stratified Cox regression analysis by refugee status was also performed. Person-time was computed from the date exactly one year after the CMD diagnosis until the date of the event for SA and DP or by right-censoring (the date of the last known information, December 31st, 2016). For UE person time was measured until January 1st of the year when > 180 annual days were registered. The proportional hazard assumption was tested by Schoenfeld residuals. We computed HRs, with 95% CI for each medication cluster using the largest cluster with overall low medication use as the reference category. The analyses were performed using R 3.6.2.

## Results

Of the 12,472 individuals who met the inclusion criteria of this study, 5429 (43.5%), were male, 1454 (11.7%) were refugees, and the mean age (SD) was 22 years (1.97).

### Baseline characteristics of refugees vs. Swedish-born

Table 1 presents baseline characteristics. Compared to refugees, Swedish-born youth had a higher number of psychiatric outpatient days, a higher prevalence of personality disorder, substance use disorder, eating disorder, ADHD, and “other” comorbid mental disorders. Moreover, refugees had a higher prevalence of “other” comorbid somatic diseases, compared to the Swedish-born youth in all clusters. There was a slightly smaller proportion of refugees with the use of antidepressants (78.9% of refugees compared to 85.9% of Swedish-born), anxiolytics (53.0 vs. 57.0%) and mood stabilizers (3.6 vs. 5.8%) while the proportion of refugees that used antipsychotics (12.2 vs. 11.7%) and sedative-hypnotics (49.1 vs. 44.8%) tended to be higher.

**Table 1:** Baseline characteristics of 12,472 young adults with a common mental disorder (CMD)

	Total (*n* = 12,472)	Refugees (*n* = 1454)	Swedish-born (*n* = 11,018)
Age (mean (SD))	22.1 (1.97)	22.44 (1.95)	22.06 (1.97)
Sex, male (%)	5429 (43.5)	613 (42.2)	4816 (43.7)
Emigration (%)	155 (1.2)	33 (2.3)	122 (1.1)
Residence region^a^ (%)			
Big cities	5320 (42.7)	626 (43.1)	4694 (42.6)
Medium-sized cities	4833 (38.8)	585 (40.2)	4248 (38.6)
Small cities/villages	2319 (18.6)	243 (16.7)	2076 (18.8)
Education^b^ (%)			
Low	5742 (46.0)	643 (44.2)	5099 (46.3)
Medium	5228 (41.9)	498 (34.3)	4730 (42.9)
High	1203 (9.6)	147 (10.1)	1056 (9.6)
Family situation (%)			
Married/living with partner without children	75 (0.6)	41 (2.8)	34 (0.3)
Married/living with partner with children	354 (2.8)	80 (5.5)	274 (2.5)
Single/divorced/separated/widowed without children	6413 (51.4)	825 (56.8)	5588 (50.7)
Single/divorced/separated/widowed with children	209 (1.7)	36 (2.5)	173 (1.6)
Children (younger than 20 years old), living at home	5420 (43.5)	471 (32.4)	4949 (44.9)
CMD type (%)			
Depression	4738 (38.0)	580 (39.9)	4158 (37.7)
Anxiety (exclude post-traumatic stress disorder)	7357 (59.0)	783 (53.9)	6574 (59.7)
Post-traumatic stress disorder	377 (3.0)	91 (6.3)	286 (2.6)
Previous healthcare or treatment, measured at one year after CMD diagnosis (%)^c^			
Inpatient care, psychiatric diagnosis			
Psychiatric inpatient stays	3001 (24.1)	346 (23.8)	2655 (24.1)
Psychiatric inpatient days	1864 (14.9)	196 (13.5)	1668 (15.1)
Inpatient care, somatic diagnosis			
Somatic inpatient stays	3488 (28.0)	468 (32.2)	3020 (27.4)
Somatic inpatient days	3010 (24.1)	394 (27.1)	2616 (23.7)
Outpatient care			
Psychiatric outpatient visits	4728 (37.9)	432 (29.7)	4296 (39.0)
Somatic outpatient visits	4359 (34.9)	571 (39.3)	3788 (34.4)
Previous diagnoses (during 3 years before the baseline)			
Personality disorder (%)	772 (6.2)	62 (4.3)	710 (6.4)
Substance use disorder (%)	1830 (14.7)	162 (11.1)	1668 (15.1)
Eating disorder (%)	599 (4.8)	38 (2.6)	561 (5.1)
Attention deficit hyperactivity disorder (%)	915 (7.3)	38 (2.6)	877 (8.0)
Autism (%)	235 (1.9)	< 10 (0.2)	232 (2.1)
Other mental disorder (%)	1749 (14.0)	190 (13.1)	1559 (14.1)
Suicide attempt (%)	1170 (9.4)	130 (8.9)	1040 (9.4)
Cancer (%)	415 (3.3)	44 (3.0)	371 (3.4)
Epilepsy (%)	124 (1.0)	19 (1.3)	105 (1.0)
Asthma (%)	336 (2.7)	15 (1.0)	321 (2.9)
Other respiratory disease (%)	1034 (8.3)	126 (8.7)	908 (8.2)
Musculoskeletal disease (%)	1393 (11.2)	164 (11.3)	1229 (11.2)
Other somatic disorder (%)	7480 (60.0)	944 (64.9)	6536 (59.3)
Psychotropic medication use, measured at one year before and after CMD (%)			
Antidepressants	10,611 (85.1)	1147 (78.9)	9464 (85.9)
Antipsychotics	1468 (11.8)	177 (12.2)	1291 (11.7)
Anxiolytics	7051 (56.5)	771 (53.0)	6280 (57.0)
Sedative-hypnotic	5645 (45.3)	714 (49.1)	4931 (44.8)
Mood stabilizer	694 (5.6)	53 (3.6)	641 (5.8)

^a^Big cities—Stockholm, Gothenburg, and Malmö; medium-sized cities—cities with more than 90,000 residents within 30 km of the city center; small cities/villages—remaining cities or villages in Sweden

^b^299 individuals have missing education information

^c^Previous healthcare or treatment was dichotomized by the mean of inpatient/outpatients stay

### Baseline characteristics by cluster

The Louvain clustering algorithm identified five clusters; the remaining individuals were classified in cluster 6 (many of these patients overwhelmingly showed non-significant patient–patient correlations after applying the disparity filter adjustment). Table S1 shows baseline characteristics by cluster membership. Anxiety disorder was highly prevalent in all clusters, compared to other types of CMD.

**Fig. 1:**
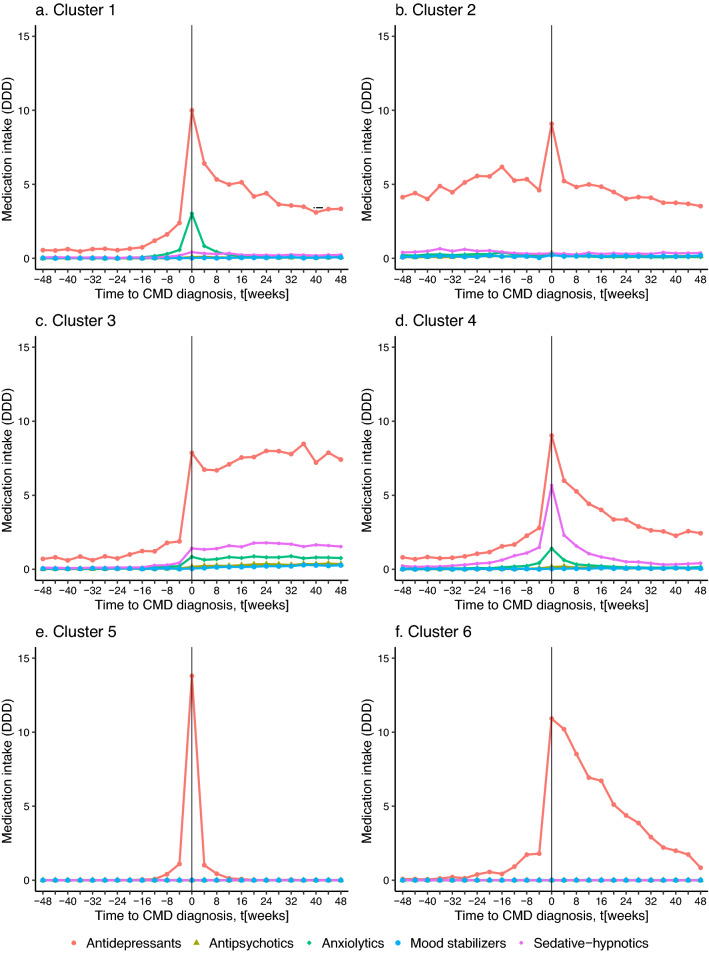
Time-dependent medication use in defined daily doses (DDD) for each medication by clusters within a two-year interval centered around the diagnosis of Common mental disorder (CMD), 12,472 individuals with a CMD diagnosis aged 18–24 from 2007 to 2013 in Sweden

Cluster 1 (briefly described as “short-term peak in antidepressants and anxiolytics at CMD diagnosis”) contained 11.1% refugees and was characterized by moderate inpatient/outpatient care, and a lower frequency of comorbid mental disorders. We observed a moderate dosage of antidepressants and a high dosage of anxiolytics in cluster 1 (Table S4). Medication use was concentrated in the weeks shortly before and after CMD (Fig. 1a).

Cluster 2 (“high antidepressants before and after CMD diagnosis”) contained 12.0% refugees. The highest dosages of antidepressants and mood stabilizers in Swedish-born youth were found in this cluster (Table S4). There were also high dosages of antidepressants sustained over the months before and after the peak around CMD diagnosis (see Fig. 1b).

Cluster 3 (“sustained increase in all medication types from time of CMD diagnosis and onwards”) comprised the highest proportion of individuals (31.2%) of which 9.2% were refugees. Of all clusters, cluster 3 had the highest mean cumulative dosages of antipsychotics, anxiolytics, and sedative-hypnotics (see Table S4). Figure 1c shows that cluster 3 is characterized by high dosages in all medications after CMD diagnosis, as well as increased dosages in antidepressants, anxiolytics, and sedative-hypnotics shortly before CMD diagnosis.

Cluster 4 (“short-term peak in antidepressants, sedative-hypnotics, and anxiolytics at CMD diagnosis”) contained 15% refugees and were characterized by a high prevalence of “other” somatic diseases. The cluster was characterized by a moderate dosage of antidepressants and a high dosage of sedative-hypnotics (Table S4). Figure 1d reveals moderate use of antidepressant, anxiolytic, and sedative-hypnotic concentrated in the weeks around CMD diagnosis for this cluster.

Cluster 5 (“very short-term peak in antidepressants at CMD diagnosis”) contained the highest proportion of refugees (18%), the lowest level of education, the least frequent hospital visits due to psychiatric diagnosis, and the lowest incidence of “other” mental disorders. Low cumulative medication use and no antipsychotic or mood stabilizer use were observed for this cluster as shown in Table S4. Figure 1e shows a sharp peak at the CMD diagnosis, and a rapid decline in antidepressant use shortly after the CMD diagnosis.

Cluster 6 (“increase in antidepressants at CMD diagnosis with gradual subsequent decline”) contained 9.7% refugees. Individuals in cluster six showed the least frequent hospital visits due to somatic diagnosis, and the lowest incidence of substance use disorder. Moderate antidepressant use and low anxiolytic and sedative-hypnotic use for this cluster are shown in Table S4. Figure 1f shows low dosages of medications, characterized by increased dosages of antidepressants around CMD diagnosis, followed by a gradual decline thereafter.

The baseline characteristics of refugee or Swedish-born youth by cluster membership are presented in Supplement table S2–S3. Compared to Swedish-born youth, young refugees had lower mean cumulative antidepressant, anxiolytic, sedative-hypnotic, and mood stabilizer use in all clusters except for antipsychotics. Additional qualitative description of each cluster is provided in the SI. The time-dependent medication intake in each cluster, by refugees vs. Swedish-born youth and anxiety disorder vs. depression, are presented in supplemental Fig. S1 and Fig. S2, respectively.

### Sensitivity analyses

We performed several sensitivity analyses to evaluate the robustness of the identified patient clusters. The clustering algorithm was repeated for refugees only, then for Swedish-born youth (See Fig. S3, S4, respectively). Further, we repeated the analysis for individuals with an anxiety disorder (Fig. S5) or depression (Fig. S6). Overall, we observed qualitatively similar medication clusters across these sensitivity analyses, with clusters 5 and 6 occasionally merging into a single cluster.

### Cox regression

Table 2 reports HRs for cluster memberships with subsequent SA, DP, and UE in crude and adjusted models in refugee and Swedish-born youth. During the mean follow-up of 4.1 years, there were 1734 (13.9%) SA, 1490 (11.9%) incidences of DP, and 1624 (13.0%) UE for all youth. Overall, young refugees had less frequent DP and more frequent periods of UE during the follow-up than the Swedish-born youth (8.7 vs. 12.4%, 24.9 vs. 11.5% of refugees vs. Swedish-born youth had DP and UE, respectively). Cluster 6 was chosen as the reference group due to low overall dosages, and substantially larger sample size than that of cluster five, the other low medication use cluster. For SA, DP, and the composite LMM, cluster three showed the highest overall HRs among young adults regardless of refugee status with CMD (HR, 95% CI 1.69 [1.34, 2.13], 2.63 [2.05, 3.38], 1.84 [1.59, 2.11], respectively). Regarding UE, cluster 5 showed the highest overall HR among young adults with CMD (HR, 95% CI 1.61[1.18, 2.18]). The association of cluster membership with all measures of LMM were similar in young refugees and Swedish-born youth with CMD. Despite only one significant estimate in the refugee youth with CMD in the stratified analysis, refugee youth showed a tendency of lower estimates in SA, DP, and the composite LMM than their Swedish-born counterparts.

**Table 2:** Association of Louvain cluster memberships to labor market marginalization (sickness absence, disability pension and unemployment) occurrences stratified by refugee status and overall (crude and multivariable Hazard ratio and 95% Confidence Intervals)

		All (*n* = 12,472)				Refugee (*n* = 1454)			Swedish-born^a^ (*n* = 11,018)		
	Cluster ID	Case/n	Incident Rate /100 year	Crude	Multivariable^b^	Case/n	Crude	Multivariable^c^	Case/n	Crude	Multivariable^c^
Sickness absence (*n* = 1734)	6^d^	80/893	1.78	1	1	< 10/87	1	1	71/806	1	1
	1	315/2450	2.72	1.53 (1.20, 1.96)	1.46 (1.15, 1.87)	44/271	1.75 (0.85, 3.59)	1.70 (0.83, 3.49)	271/2179	1.50 (1.15, 1.95)	1.45 (1.12, 1.89)
	2	286/2039	2.88	1.62 (1.27, 2.08)	1.55 (1.21, 1.99)	27/244	1.16 (0.55, 2.47)	1.08 (0.51, 2.29)	259/1795	1.69 (1.30, 2.20)	1.62 (1.25, 2.11)
	3	584/3887	3.17	1.78 (1.41, 2.25)	1.69 (1.34, 2.13)	54/359	1.55 (0.77, 3.15)	1.57 (0.77, 3.17)	530/3528	1.81 (1.41, 2.32)	1.71 (1.33, 2.19)
	4	431/2784	3.20	1.80 (1.42, 2.28)	1.68 (1.32, 2.13)	62/417	1.51 (0.75, 3.05)	1.48 (0.73, 2.97)	369/2367	1.85 (1.43, 2.38)	1.71 (1.33, 2.21)
	5^e^	38/419	1.77	1.00 (0.68, 1.47)	*	< 10/76	1.04 (0.40, 2.70)	*	30/343	0.98 (0.64, 1.50)	0.99 (0.65, 1.52)
Disability pension (*n* = 1490)	6	68/893	1.52	1	1	< 10/87	1	1	61 /806	1	1
	1	170/2450	1.43	0.93 (0.7, 1.23)	0.99 (0.75, 1.32)	11/271	0.50 (0.19, 1.29)	0.54 (0.21, 1.40)	159/2179	0.98 (0.73, 1.32)	1.05 (0.78, 1.41)
	2	265/2039	2.73	1.78 (1.36, 2.32)	1.85 (1.42, 2.42)	22/244	1.14 (0.49, 2.67)	1.19 (0.51, 2.78)	243/1795	1.87 (1.41, 2.48)	1.92 (1.45, 2.54)
	3	696/3887	4.03	2.57 (2.01, 3.30)	2.63 (2.05, 3.38)	55/359	2.00 (0.91, 4.39)	1.97 (0.90, 4.33)	641/3528	2.64 (2.03, 3.43)	2.71 (2.08, 3.52)
	4	267/2784	1.94	1.27 (0.98, 1.66)	1.32 (1.01, 1.72)	28/417	0.82 (0.36, 1.87)	0.81 (0.35, 1.86)	239/2367	1.36 (1.03, 1.80)	1.39 (1.05, 1.84)
	5	24/419	1.13	0.74 (0.47, 1.18)	*	< 10/76	0.47 (0.12, 1.81)	*	21/343	0.81 (0.49, 1.33)	*
Unemployment (*n* = 1624)	6	93/893	2.07	1	1	18/87	1	1	75/806	1	1
	1	286/2450	2.46	1.19 (1.19, 1.50)	1.21 (0.96, 1.53)	49/271	0.91 (0.53, 1.57)	0.96 (0.56, 1.65)	237/806	1.24 (0.96, 1.61)	1.28 (0.99, 1.66)
	2	290/2039	2.91	1.41 (1.41, 1.78)	1.39 (1.10, 1.75)	69/244	1.50 (0.90, 2.53)	1.53 (0.91, 2.57)	221/1795	1.35 (1.04, 1.76)	1.35 (1.04, 1.75)
	3	498/3887	2.64	1.28 (1.28, 1.59)	1.29 (1.03, 1.61)	95/359	1.31 (0.79, 2.16)	1.30 (0.78, 2.15)	403/3528	1.28 (1.00, 1.63)	1.29 (1.01, 1.65)
	4	383/2784	2.81	1.36 (1.36, 1.7)	1.25 (1.00, 10.57)	109/417	1.30 (0.79, 2.14)	1.27 (0.77, 2.10)	274/2367	1.28 (0.99, 1.65)	1.26 (0.97, 1.62)
	5	74/419	3.69	1.78 (1.78, 2.42)	1.61 (1.18, 2.18)	22/76	1.49 (0.80, 2.77)	1.46 (0.78, 2.72)	52/343	1.71 (1.20, 2.44)	1.67 (1.17, 2.37)
Labor market marginalization (*n* = 4206)	6	221/893	5.54	1	1	18/87	1	1	75/806	1	1
	1	664/2450	6.46	1.16 (0.99, 1.35)	1.18 (1.01, 1.37)	92/271	0.95 (0.63, 1.42)	0.97 (0.65, 1.45)	572/2179	1.19 (1.01, 1.40)	1.21 (1.03, 1.43)
	2	722/2039	8.77	1.57 (1.35, 1.83)	1.55 (1.34, 1.81)	103/244	1.24 (0.83, 1.84)	1.24 (0.83, 1.85)	619/1795	1.62 (1.37, 1.90)	1.60 (1.36, 1.89)
	3	1524/3887	10.40	1.85 (1.61, 2.13)	1.84 (1.59, 2.11)	175/359	1.50 (1.03, 2.19)	1.47 (1.01, 2.15)	1349/3528	1.91 (1.64, 2.22)	1.90 (1.63, 2.21)
	4	957/2784	8.33	1.49 (1.29, 1.73)	1.43 (1.24, 1.66)	179/417	1.22 (0.84, 1.78)	1.2 (0.82, 1.76)	778/2367	1.51 (1.29, 1.77)	1.47 (1.25, 1.72)
	5	118/419	6.49	1.17 (0.94, 1.46)	1.12 (0.90, 1.40)	28/76	1.01 (0.61, 1.67)	0.99 (0.60, 1.65)	90/343	1.16 (0.90, 1.49)	1.13 (0.88, 1.46)

Sickness absence was defined as greater than 90 annual net days; unemployment was defined as greater than 180 annual days; labor market marginalization was defined as a composite variable of at least one occurrence of sickness absences, disability pension or unemployment

^a^Swedish-born indicates a native-born Swede with parents who were born in Sweden

^b^Multivariable model in the overall population, adjusted for education, refugee status, previous sickness absence spells of greater than 90 days, previous long-term unemployment of greater than 180 days

^c^Multivariable model in Swedish-born and refugees, adjusted for education, previous sickness absence spells of greater than 90 days, previous long-term unemployment

^d^Cluster 6 was chosen as the reference group due to low overall dosages, and substantially larger sample size than that of cluster 5, the other low medication use cluster

^e^Cluster 5 has a low number of sickness absences and disability pensions for the multivariable model

## Discussion

We identified six distinct groups of patients characterized by specific medication use patterns over time. First, we found a group of patients with a brief peak in antidepressant and anxiolytic use at CMD diagnosis. Second, a group showed high antidepressant use before and after CMD diagnosis. Third was a cluster with a sustained increase in all medication types from the time of CMD diagnosis onwards. Fourth, we identified a group with a short-term peak in antidepressants, sedative-hypnotics and anxiolytics at CMD diagnosis. The fifth group was patients with a very brief peak in antidepressants at CMD diagnosis. Sixth and finally, we found patients with an increase in antidepressants at CMD diagnosis followed by a slower decline. Compared to the Swedish-born youth, we found that young refugees had a general tendency towards lower medication use, except for antipsychotics in clusters 2 and 3. Cluster 3 showed the highest elevated risk of SA and DP, while cluster 5 showed the highest elevated risk of UE. The association of cluster membership with LMM was similar in both refugees and Swedish-born youth with CMD.

Overall, psychotropic medication use among refugee youths was lower than among their Swedish-born counterparts. This finding is in line with other studies examining the relationship between refugee status and psychotropic medications [14, 16, 19–21]. Lower amounts of dispensed psychotropic medications were reported among newly resettled refugees in Sweden, compared to native Swedish-born, based on an analysis of over one million young adults [20, 24]. Moreover, several studies from our research group have found lower initiation, adherence, and usage of antidepressants among young refugee youth with CMD than among their Swedish-born counterparts. [14, 16, 21]

The difference between refugee and Swedish-born young adults with CMD in terms of psychotropic medication use could be attributed to several factors. First, low psychotropic medication use may reflect lower access to psychiatric care due to limited knowledge of the healthcare system, language barriers, and acculturation in the host country [20]. Moreover, lower mental health literacy and higher stigmatization of mental disorders among refugees may lead to poor medication adherence or poor initiation of treatment after CMD diagnosis [22–24]. Young adult refugees showed also lower mental health literacy than the native-born in Germany, Australia, and Sweden [23–25]. The cultural background of refugees may influence attitudes and beliefs toward psychotropic medication and mental disorders [22–24]. For instance, mental disorders were associated with feelings of being ashamed and socially unaccepted in some Asian cultures [26]. Social distance towards individuals with a mental disorder was also found in Nigeria [27]. Sudanese refugees in Australia perceived supernatural forces to be the cause of mental disorders [24]. Furthermore, several studies showed that spiritual coping strategies are preferred among some groups of refugees [24, 28]. The mentioned factors could thus impede refugees’ willingness to engage in treatment and comply with pharmacological treatment.

Higher use of antipsychotics in refugees than in their Swedish-born counterparts was observed, contrary to a general tendency of the Swedish-born showing higher medication use than refugees in most of the other considered medication categories. Antipsychotic medications are used not only as a first-line treatment for psychosis, such as schizophrenia but also in combination with selective serotonin reuptake inhibitors (SSRIs) to treat post-traumatic stress disorder (PTSD) [29–31]. A previous study examining the psychiatric utilization among refugee, non-refugee migrants, and Swedish-born youth also suggested higher use of psychiatric care among refugees due to PTSD [32]. The higher prevalence of PTSD and psychotic disorders among young refugees, which may have led to the higher prescription rates of antipsychotics, was potentially due to the traumas, stressors, and social adversities arising from forced migration. [33, 34]

This study identified six clusters with different prescription patterns. Each cluster showed distinguishable characteristics and the dynamic of medication use centered around CMD diagnosis. The clusters also showed different risks of subsequent LMM. Cluster 3, characterized by a sustained increase in all medication types from the time of CMD diagnosis onward, showed the highest elevated risk of SA, DP, and the composite measure of LMM. Patient cluster 5, characterized by a very short-term peak in antidepressants at CMD diagnosis, was associated with the highest elevated risk of UE. We observed similar associations between cluster membership and LMM in refugee and Swedish-born youth.

While cluster 5 (“very short-term peak in antidepressants at CMD diagnosis”) did not have a substantially increased LMM risk compared to the reference cluster 6, the former did have a higher risk of UE. This finding could be attributed to the higher proportion of refugees in cluster 5. Lower educational attainment, inadequate language proficiency, and discrimination among young refugees impose more difficulties to obtain and keep a job [29]. Job instability and financial hardship among young refugees is a potential stressor for exacerbating the symptoms of CMDs [8], thereby further lowering the capability to engage in the labor market. Moreover, a high proportion of individuals in cluster five residing in small cities/villages and seeking fewer psychiatry treatments was observed in table S1. This geographic difference hinders long-distance commutes and increases barriers to accessing healthcare. However, previous studies on the role of rurality in healthcare utilization were inconsistent [39, 40]. Further, the sharp decline in antidepressant use within four weeks after CMD diagnosis in cluster five could be indicative of poor compliance with treatment among the refugee youth [17, 35]. As the World Health Organization treatment guidelines recommend at least 9–12 months of pharmacological treatment for adults with the depressive disorder [35], the rapid lowering of dosages is inconsistent with the treatment guideline. The observed pattern may thus reflect early medication discontinuation or noncompliance with the treatment. Young refugees might have a lower tendency to comply with the treatment, given the uncertainty about the efficacy of pharmaceutical treatment among refugees [41]. In turn, poor adherence to antidepressants may worsen the severity of mental disorders and further marginalize refugee youth from the labor market. Providing vocational training, monitoring medication adherence, and delivering adequate healthcare access particularly in rural area might be helpful strategies to lower the risk of premature labor market exit.

We found consistently low dosages before CMD and high dosages after CMD across all medication categories in Cluster 3 (“sustained increase in all medication types from time of CMD diagnosis”). This pattern may reflect undertreated individuals with CMD before the diagnosis. While the study population included only incident cases of CMD, it should be noted that the CMD diagnoses were measured in specialized health care only. As CMDs often have an early age of onset, it is likely that individuals had health care contacts within primary health care sometime before their first contact with specialized health care. Individuals in cluster 3 might have a lower inclination to health care seeking, which then led to higher severity of the mental disorders, particularly at a young age [36]. Furthermore, the higher levels of DDDs after the CMD diagnosis might be an indication of the increasing severity of the CMD symptoms and/or a poor prognosis necessitating higher dosages of psychiatric medication. This medication pattern corresponded to the highest elevated risk of SA and DP in our study. Workability is naturally strongly affected during periods with the severe symptoms of CMD (i.e. mood instability, cognitive impairment and sleep deprivation). [37]

### Strengths and limitations

The strengths of the current study include a large study population with information on national coverage and data of good quality [38]. Moreover, the long follow-up duration, the prospective design, the extensive dispensing information obtained from the Prescribed Drug Register and the use of an advanced statistical model to classify individuals are clear strengths of the study. Our study has also limitations needed to be mentioned. First, we could not evaluate the relation of several cluster memberships with DP or sickness absences among refugees due to the low case numbers. Additionally, the number of young adults in need of CMD treatment may be underestimated. We could only collect information from the specialized healthcare setting' the information from the primary care setting is unavailable on a national level. The majority of individuals with CMD are, however, treated in the primary health care setting. Moreover, changes in the distributions of countries of origin in newly settled refugees after 2016 are not captured in our dataset. Further, rates of drug dispensing are not equal to medication utilization by patients. Finally, our study may not be generalizable to countries with different health care and social insurance systems, such as fee-based systems.

## Conclusions

Young adult refugees with CMD showed lower psychotropic medication use compared to their Swedish-born counterparts over a two-year exposure period. The medication use patterns, classified by six distinguishable cluster memberships, were differentially associated with LMM; the association was similar in refugee and Swedish-born youth. However, there was a higher fraction of refugees in a high-risk cluster for UE with rapidly declining medication use, compatible with the premature discontinuation of treatment. Our findings suggest that an examination of temporal medication use patterns might be a helpful tool for identifying high-risk patient groups.

### Supplementary Information

Below is the link to the electronic supplementary material.

Supplementary file1 (PDF 508 KB)

## Data Availability

The dataset is subject to licenses and restrictions due to its highly sensitive microdata. Microdata cannot be made publicly available according to several laws. Inquiry to access the dataset could contact Karolinska Institutet's Division of Insurance Medicine.
