# Supplementary material for: Association of medication clusters and subsequent labor market marginalization in refugee and Swedish-born young adults with common mental disorders in Sweden
Source: Eur Child Adolesc Psychiatry. 2023 Apr 28;33(3):897–907. doi: 10.1007/s00787-023-02214-8 (PMC10894142; doi:10.1007/s00787-023-02214-8)
Supplement: Supplementary file 1 — Supplementary file1 (PDF 508 KB) [file 787_2023_2214_MOESM1_ESM.pdf]

Title: Association of medication clusters and subsequent labor market marginalization in refugee and Swedish-born young adults with common mental disorder in Sweden

Chen J,[1,2,3] Mittendorfer-Rutz E,[3] Taipale H,[3,4,5] Rahman S,[3] Niederkrotenthaler T,[6,7] Klimek P[1,2 *]

[1] Section for Science of Complex Systems, CeMSIIS
Medical University of Vienna, Vienna, Austria.
[2] Complexity Science Hub Vienna, Vienna, Austria.
[3] Division of Insurance Medicine, Department of Clinical Neuroscience,
Karolinska Institutet, SE-171 77, Stockholm, Sweden.
[4] Niuvanniemi Hospital, Kuopio, Finland.
[5] University of Eastern Finland, School of Pharmacy, Kuopio, Finland.
[6] Unit Suicide Research and Mental Health Promotion, Department of Social and Preventive Medicine,
Centre for Public Health, Medical University of Vienna, Vienna, Austria.
[7] Wiener Werkstätte for Suicide Research, Vienna, Austria.

* **Corresponding author: Peter Klimek**

Section for Science of Complex Systems, CeMSIIS
Medical University of Vienna, Vienna, Austria
peter.klimek@meduniwien.ac.at

Supplementary Information

SI Statistical Methods

Louvain clustering algorithm requires no specification of the number of clusters and their expected structure but rather learns these properties from the data. The mean cumulative medication dosage of each medication category for each patient was represented as a time series spanning over the exposure period. For each individual we computed the weekly cumulative DDD dispensed in each medication category. We constructed a patient–patient similarity scores by correlating these time series. That is, for medication category x we computed the Pearson correlation between the cumulative time series of two individuals i and j, giving the Pearson correlation $\rho_x(i,j)$. Note that by definition of the Pearson correlation coefficient, the cumulative time series of medications are effectively rescaled. Hence, one can think of them as time series for all individuals and medication use starts at zero and increases to a maximum value at the end of the observation period. Individuals, for which all medications occur during a short time interval, the resulting curves will look similar to step functions. The curves for individuals with regularly dispensed medications will be smoother and approach a straight line if the same dosages were dispensed in each interval over the two-year exposure period. The Pearson correlation coefficient, therefore, measures whether two patients have similarly shaped curves (smooth or step-like, and if there is a step, whether this step occurs at similar time points). We then computed the Euclidean norm of $\rho_x(i,j)$ over all categories x to obtain the overall patient–patient similarity score. High similarity further indicates that patients show similar cumulative time series in more than one medication category. Non-significant patient–patient correlations were removed using a disparity filter[1]with a threshold value of 0.28. Individuals without significant correlations with any other patient were grouped into a separate cluster. The identified medication cluster memberships were formalized as a categorical cluster membership variable. To summarize, in our approach each individual was assigned to a cluster category according to her/his medication pattern without pre-specifying these patterns.

Reference

1. Serrano MA, Boguna M, Vespignani A. Extracting the multiscale backbone of complex weighted networks. *Proceedings of the National Academy of Sciences*. 2009/04/08/ 2009:6483-6488 IS -16 VL -106. doi:10.1073/pnas.0808904106

SI Cluster Descriptions

Cluster membership	Short label	Additional descriptions
**1**	Short-term peak in antidepressant and anxiolytic use at CMD diagnosis.	Antidepressant dosage started to rise eight weeks before the CMD diagnosis, then increased sharply. Antidepressant and anxiolytic use peaked at around CMD diagnosis and dropped rapidly after the CMD diagnosis. Low anxiolytic and sedative-hypnotic dosage, and no antipsychotic or mood stabilizer dosage was observed during the two-year exposure period.
**2**	High antidepressants before and after CMD diagnosis	The dosage of antidepressants before CMD diagnosis was rather high. Use of antidepressants, anxiolytics, and sedative-hypnotics sharply rose four weeks before the CMD diagnosis. Dosage of mood stabilizers and antipsychotics steadily increased after the CMD diagnosis.
**3**	Sustained increase in all medication types from time of CMD diagnosis onwards	Antidepressant dosage increased sharply four weeks before the CMD diagnosis. The highest increment peaked around the time of the CMD diagnosis, then rapidly declined. Anxiolytics and sedative-hypnotics have several high increments during the two-year period studied. The medication dosage remained high after the CMD diagnosis. Low dosage of mood stabilizers and antipsychotics across the exposure period were shown.
**4**	Short-term peak in antidepressants, sedative-hypnotics and anxiolytics at CMD diagnosis.	During the one year before the CMD diagnosis, antidepressants, anxiolytics, and sedative-hypnotics trended upward and peaked at around the time of CMD diagnosis. The medication dosage declined after the CMD diagnosis. Low dosage of antipsychotics and mood stabilizers were observed, with the highest increment of antipsychotics occurring around four weeks after the CMD diagnosis.
**5**	Very short-term peak in antidepressants at CMD diagnosis.	Antidepressants increased rapidly four weeks before the CMD diagnosis and peaked at CMD diagnosis. The dosage of antidepressants declined rapidly within four weeks after the CMD diagnosis. Individuals in the cluster showed low dosage of anxiolytics and sedative-hypnotics, while no dispensing of antipsychotic and mood stabilizers was observed.
**6**	Increase in antidepressants at CMD diagnosis with gradual subsequent decline	The dosage of antidepressants started to increase weeks before the CMD diagnosis, then sharply increased four weeks before the CMD diagnosis and peaked at around the CMD diagnosis. The dosage gradually declined after the CMD diagnosis. Low dosages of anxiolytics and sedative-hypnotics, but no dispensing of antipsychotic and mood stabilizers were observed.

Supplementary Tables

Table S1 Baseline characteristic of 12,472 young adults with common mental disorder (CMD) according to Louvain cluster memberships

	**1**	**2**	**3**	**4**	**5**	**6**	p[1]
n	2450	2039	3887	2784	419	893	
Age (mean (SD))	22.2 (1.9)	22.2 (2.0)	22.0 (2.0)	22.2 (2.0)	21.9 (2.0)	21.7 (2.0)	< 0.001
Sex, male (%)	1036 (42.3)	858 (42.1)	1637 (42.1)	1252 (45.0)	210 (50.1)	436 (48.8)	< 0.001
Refugee (%)	271 (11.1)	244 (12.0)	359 (9.2)	417 (15.0)	76 (18.1)	87 (9.7)	< 0.001
**Emigration** (%)	36 (1.5)	16 (0.8)	47 (1.2)	40 (1.4)	<10 (1.0)	12 (1.3)	0.330
**Residence region[2] (%)**							< 0.001
Big cities	1047 (42.7)	905 (44.4)	1698 (43.7)	1159 (41.6)	130 (31.0)	381 (42.7)	
Medium-sized cities	968 (39.5)	760 (37.3)	1468 (37.8)	1117 (40.1)	180 (43.0)	340 (38.1)	
Small cities/villages	435 (17.8)	374 (18.3)	721 (18.5)	508 (18.2)	109 (26.0)	172 (19.3)	
**Education[3] (%)**							< 0.001
Low	1018 (41.6)	943 (46.2)	1833 (47.2)	1269 (45.6)	235 (56.1)	444 (49.7)	
Medium	1136 (46.4)	831 (40.8)	1580 (40.6)	1179 (42.3)	147 (35.1)	355 (39.8)	
High	258 (10.5)	218 (10.7)	379 (9.8)	241 (8.7)	32 (7.6)	75 (8.4)	
**Family situation (%)**							< 0.001
Married/living with partner without children	13 (0.5)	17 (0.8)	17 (0.4)	21 (0.8)	<10 (0.2)	<10 (0.7)	
Married/living with partner with children	68 (2.8)	50 (2.5)	105 (2.7)	94 (3.4)	17 (4.1)	20 (2.2)	
Single/divorced/separated/widowed without children	1271 (51.9)	1121 (55.0)	1932 (49.7)	1503 (54.0)	203 (48.4)	383 (42.9)	
Single/divorced/separated/widowed with children	47 (1.9)	33 (1.6)	57 (1.5)	52 (1.9)	10 (2.4)	10 (1.1)	
Children (younger than 20) living at home	1051 (42.9)	818 (40.1)	1775 (45.7)	1114 (40.0)	188 (44.9)	474 (53.1)	
**CMD type (%)**							< 0.001
Depression	808 (33.0)	710 (34.8)	1482 (38.1)	1071 (38.5)	231 (55.1)	436 (48.8)	
Anxiety disorder (excluding post-traumatic stress disorder)	1603 (65.4)	1245 (61.1)	2292 (59.0)	1605 (57.7)	177 (42.2)	435 (48.7)	
Post-traumatic stress disorder	39 (1.6)	84 (4.1)	113 (2.9)	108 (3.9)	11 (2.6)	22 (2.5)	
**Previous healthcare or treatment measured one year after CMD diagnosis[4]**							
**Inpatient care, psychiatric diagnosis**							
Psychiatric inpatient stays (mean (SD))	417 (17.0)	445 (21.8)	1172 (30.2)	771 (27.7)	57 (13.6)	139 (15.6)	< 0.001
Psychiatric inpatient days (mean (SD))	215 (8.8)	275 (13.5)	813 (20.9)	470 (16.9)	23 (5.5)	68 (7.6)	< 0.001
**Inpatient care, somatic diagnosis**							

Somatic inpatient stays (mean (SD))	601 (24.5)	599 (29.4)	1166 (30.0)	839 (30.1)	97 (23.2)	186 (20.8)	< 0.001
Somatic inpatient days (mean (SD))	515 (21.0)	514 (25.2)	1027 (26.4)	711 (25.5)	80 (19.1)	163 (18.3)	< 0.001
**Outpatient care**							
Psychiatric outpatient visits (mean (SD))	757 (30.9)	688 (33.7)	2088 (53.7)	897 (32.2)	52 (12.4)	246 (27.5)	< 0.001
Somatic outpatient (mean (SD))	759 (31.0)	806 (39.5)	1441 (37.1)	1001 (36.0)	116 (27.7)	236 (26.4)	< 0.001
**Previous diagnoses (during 3 years before the baseline)**							
Personality disorder (%)	113 (4.6)	137 (6.7)	335 (8.6)	150 (5.4)	<10 (2.1)	28 (3.1)	< 0.001
Substance use disorder (%)	272 (11.1)	340 (16.7)	657 (16.9)	430 (15.4)	47 (11.2)	84 (9.4)	< 0.001
Eating disorder (%)	89 (3.6)	127 (6.2)	222 (5.7)	111 (4.0)	17 (4.1)	33 (3.7)	< 0.001
Attention deficit hyperactivity disorder (%)	118 (4.8)	197 (9.7)	326 (8.4)	204 (7.3)	24 (5.7)	46 (5.2)	< 0.001
Autism (%)	26 (1.1)	53 (2.6)	86 (2.2)	46 (1.7)	< 10 (0.7)	21 (2.4)	0.001
Other mental disorder (%)	253 (10.3)	349 (17.1)	647 (16.6)	369 (13.3)	39 (9.3)	92 (10.3)	< 0.001
Suicide attempt (%)	161 (6.6)	189 (9.3)	471 (12.1)	279 (10.0)	22 (5.3)	48 (5.4)	< 0.001
Cancer (%)	85 (3.5)	69 (3.4)	142 (3.7)	78 (2.8)	15 (3.6)	26 (2.9)	0.492
Epilepsy (%)	17 (0.7)	37 (1.8)	49 (1.3)	19 (0.7)	< 10 (0.2)	< 10 (0.1)	< 0.001
Asthma(%)	61 (2.5)	59 (2.9)	108 (2.8)	72 (2.6)	12 (2.9)	24 (2.7)	0.965
Other respiratory disease (%)	194 (7.9)	170 (8.3)	341 (8.8)	241 (8.7)	26 (6.2)	62 (6.9)	0.247
Musculoskeletal disease (%)	263 (10.7)	222 (10.9)	442 (11.4)	337 (12.1)	46 (11.0)	83 (9.3)	0.267
Other somatic disorder (%)	1415 (57.8)	1208 (59.2)	2411 (62.0)	1766 (63.4)	216 (51.6)	464 (52.0)	< 0.001

[1] Differences between cluster memberships were tested by t test or chi-square test for continuous or categorical variable, respectively
[2] Big cities—Stockholm, Gothenburg, and Malmö; medium-sized cities—cities with more than 90,000 residents within 30 kilometers of the city center; small cities/villages— remaining cities or villages in Sweden.
[3] 299 individuals have missing education information
[4] Previous healthcare or treatment was dichotomized by the mean of inpatient/outpatient stays.

Table S2 Baseline characteristic of 11,018 Swedish-born[1] young adults with common mental disorder (CMD) according to cluster membership						
	**1**	**2**	**3**	**4**	**5**	**6**
n	2179	1795	3528	2367	343	806
Age (mean (SD))	22.1 (1.9)	22.2 (2.0)	22.0 (2.0)	22.2 (2.0)	21.8 (2.0)	21.6 (2.0)
Sex, male (%)	944 (43.3)	744 (41.4)	1478 (41.9)	1066 (45.0)	181 (52.8)	403 (50.0)
**Emigration** (%)	29 (1.3)	11 (0.6)	42 (1.2)	27 (1.1)	<10 (0.9)	10 (1.2)
**Residence region[2] (%)**						
Big cities	922 (42.3)	808 (45.0)	1551 (44.0)	977 (41.3)	96 (28.0)	340 (42.2)
Medium-sized cities	866 (39.7)	648 (36.1)	1326 (37.6)	945 (39.9)	158 (46.1)	305 (37.8)
Small cities/villages	391 (17.9)	339 (18.9)	651 (18.5)	445 (18.8)	89 (25.9)	161 (20.0)
**Education[3] (%)**						
Low	910 (41.8)	828 (46.1)	1664 (47.2)	1098 (46.4)	195 (56.9)	404 (50.1)
Medium	1028 (47.2)	749 (41.7)	1467 (41.6)	1035 (43.7)	125 (36.4)	1028 (47.2)
High	224 (10.3)	191 (10.6)	347 (9.8)	204 (8.6)	23 (6.7)	224 (10.3)
**Family situation (%)**						
Married/living with partner without children	< 10 (0.1)	< 10 (0.5)	11 (0.3)	< 10 (0.3)	< 10 (0.0)	< 10 (0.4)
Married/living with partner with children	51 (2.3)	38 (2.1)	88 (2.5)	71 (3.0)	11 (3.2)	15 (1.9)
Single/divorced/separated/widowed without children	1132 (52.0)	975 (54.3)	1728 (49.0)	1252 (52.9)	159 (46.4)	342 (42.4)
Single/divorced/separated/widowed with children	40 (1.8)	27 (1.5)	52 (1.5)	39 (1.6)	7 (2.0)	< 10 (1.0)
Children (younger than 20) living at home	953 (43.7)	746 (41.6)	1649 (46.7)	997 (42.1)	166 (48.4)	438 (54.3)
**CMD type (%)**						
Depression	716 (32.9)	624 (34.8)	1353 (38.4)	897 (37.9)	181 (52.8)	387 (48.0)
Anxiety disorder(exclude post-traumatic stress disorder)	1432 (65.7)	1108 (61.7)	2088 (59.2)	1391 (58.8)	154 (44.9)	401 (49.8)
Post-traumatic stress disorder	31 (1.4)	63 (3.5)	87 (2.5)	79 (3.3)	< 10 (2.3)	18 (2.2)
**Previous healthcare or treatment measured one year after CMD diagnosis[4]**						
**Inpatient care, psychiatric diagnosis**						
Psychiatric inpatient stays (mean (SD))	376 (17.3)	384 (21.4)	1058 (30.0)	667 (28.2)	46 (13.4)	124 (15.4)
Psychiatric inpatient days (mean (SD))	196 (9.0)	238 (13.3)	742 (21.0)	413 (17.4)	19 (5.5)	60 (7.4)
**Inpatient care, somatic diagnosis**						
Somatic inpatient stays (mean (SD)	527 (24.2)	515 (28.7)	1041 (29.5)	703 (29.7)	76 (22.2)	527 (24.2)
Somatic inpatient days (mean (SD))	452 (20.7)	445 (24.8)	923 (26.2)	598 (25.3)	61 (17.8)	452 (20.7)
**Outpatient care**						

Psychiatric outpatient visits (mean (SD))	696 (31.9)	622 (34.7)	1922 (54.5)	787 (33.2)	42 (12.2)	696 (31.9)
Somatic outpatient visits (mean (SD))	660 (30.3)	707 (39.4)	1296 (36.7)	833 (35.2)	87 (25.4)	660 (30.3)
**Previous diagnoses (during 3 years before the baseline)**						
Personality disorder (%)	105 (4.8)	124 (6.9)	315 (8.9)	133 (5.6)	< 10 (2.3)	25 (3.1)
Substance use disorder (%)	261 (12.0)	305 (17.0)	596 (16.9)	391 (16.5)	43 (12.5)	72 (8.9)
Eating disorder (%)	82 (3.8)	123 (6.9)	209 (5.9)	101 (4.3)	14 (4.1)	32 (4.0)
Attention deficit hyperactivity disorder (%)	115 (5.3)	187 (10.4)	315 (8.9)	194 (8.2)	23 (6.7)	43 (5.3)
Autism (%)	25 (1.1)	53 (3.0)	84 (2.4)	46 (1.9)	< 10 (0.9)	21 (2.6)
Other mental disorder (%)	227 (10.4)	308 (17.2)	588 (16.7)	324 (13.7)	30 (8.7)	82 (10.2)
Suicide attempt (%)	143 (6.6)	168 (9.4)	438 (12.4)	238 (10.1)	14 (4.1)	39 (4.8)
Cancer (%)	74 (3.4)	64 (3.6)	133 (3.8)	65 (2.7)	12 (3.5)	23 (2.9)
Epilepsy (%)	17 (0.8)	30 (1.7)	42 (1.2)	14 (0.6)	< 10 (0.3)	< 10 (0.1)
Asthma (%)	57 (2.6)	55 (3.1)	104 (2.9)	72 (3.0)	10 (2.9)	23 (2.9)
Other respiratory disease (%)	172 (7.9)	149 (8.3)	303 (8.6)	207 (8.7)	19 (5.5)	58 (7.2)
Musculoskeletal disease (%)	233 (10.7)	195 (10.9)	399 (11.3)	291 (12.3)	39 (11.4)	72 (8.9)
Other somatic disorder (%)	1247 (57.2)	1048 (58.4)	2168 (61.5)	1489 (62.9)	172 (50.1)	412 (51.1)

[1] Swedish-born indicates an native-born Swedes with parents who were born in Sweden
[2] Big cities—Stockholm, Gothenburg, and Malmö; medium-sized cities—cities with more than 90,000 residents within 30 kilometers of the city center; small cities/villages— remaining cities or villages in Sweden.
[3] 133 individuals have missing education information
[4] Previous healthcare or treatment was dichotomized by the mean of inpatient/outpatient stays.

Table S3 Baseline characteristic of 1,454 refugee young adults with common mental disorder (CMD) according to cluster memberships

	1	2	3	4	5	6
n	271	244	359	417	76	87
Age (mean (SD))	22.5 (1.9)	22.5 (1.9)	22.3 (2.0)	22.5 (1.9)	22.4 (2.1)	22.2 (2.2)
Sex, male (%)	92 (33.9)	114 (46.7)	159 (44.3)	186 (44.6)	29 (38.2)	33 (37.9)
**Emigration** (%)	< 10 (2.6)	< 10 (2.0)	< 10 (1.4)	13 (3.1)	< 10 (1.3)	< 10 (2.3)
**Residence region[1] (%)**						
Big cities	125 (46.1)	97 (39.8)	147 (40.9)	182 (43.6)	34 (44.7)	41 (47.1)
Medium-sized cities	102 (37.6)	112 (45.9)	142 (39.6)	172 (41.2)	22 (28.9)	35 (40.2)
Small cities/villages	44 (16.2)	35 (14.3)	70 (19.5)	63 (15.1)	20 (26.3)	11 (12.6)
**Education[2] (%)**						

Low	108 (39.9)	115 (47.1)	169 (47.1)	171 (41.0)	40 (52.6)	40 (46.0)
Medium	108 (39.9)	82 (33.6)	113 (31.5)	144 (34.5)	22 (28.9)	29 (33.3)
High	34 (12.5)	27 (11.1)	32 (8.9)	37 (8.9)	< 10 (11.8)	< 10 (9.2)
**Family situation (%)**						
Married/living with partner without children	10 (3.7)	< 10 (3.3)	< 10 (1.7)	13 (3.1)	< 10 (1.3)	< 10 (3.4)
Married/living with partner with children	17 (6.3)	12 (4.9)	17 (4.7)	23 (5.5)	< 10 (7.9)	< 10 (5.7)
Single/divorced/separated/widowed without children	139 (51.3)	146 (59.8)	204 (57.0)	251 (60.2)	44 (57.9)	41 (47.1)
Single/divorced/separated/widowed with children	< 10 (2.6)	< 10 (2.5)	< 10 (1.4)	13 (3.1)	< 10 (3.9)	< 10 (2.3)
Children (younger than 20) living at home	98 (36.2)	72 (29.5)	126 (35.2)	117 (28.1)	22 (28.9)	36 (41.4)
**CMD type (%)**						
Depression	92 (33.9)	86 (35.2)	129 (35.9)	174 (41.7)	50 (65.8)	49 (56.3)
Anxiety disorder (excluding post-traumatic stress disorder)	171 (63.1)	137 (56.1)	204 (56.8)	214 (51.3)	23 (30.3)	34 (39.1)
Post-traumatic stress disorder	< 10 (3.0)	21 (8.6)	26 (7.2)	29 (7.0)	< 10 (3.9)	< 10 (4.6)
**Previous healthcare or treatment measured at one year after CMD diagnosis[3]**						
**Inpatient care, psychiatric diagnosis**						
Psychiatric inpatient visits (mean (SD))	41 (15.1)	61 (25.0)	114 (31.8)	104 (24.9)	11 (14.5)	15 (17.2)
Psychiatric inpatient days (mean (SD))	19 (7.0)	37 (15.2)	71 (19.8)	57 (13.7)	< 10 (5.3)	< 10 (9.2)
**Inpatient care, somatic diagnosis**						
Somatic inpatient visits (mean (SD))	74 (27.3)	84 (34.4)	125 (34.8)	136 (32.6)	21 (27.6)	28 (32.2)
Somatic inpatient days (mean (SD))	63 (23.2)	69 (28.3)	104 (29.0)	113 (27.1)	19 (25.0)	26 (29.9)
**Outpatient care**						
Psychiatric outpatient visits (mean (SD))	61 (22.5)	66 (27.0)	166 (46.2)	110 (26.4)	10 (13.2)	19 (21.8)
Somatic outpatient visits (mean (SD))	99 (36.5)	99 (40.6)	145 (40.4)	168 (40.3)	29 (38.2)	31 (35.6)
**Previous diagnoses (during 3 years before the baseline)**						
Personality disorder (%)	< 10 (3.0)	13 (5.3)	20 (5.6)	17 (4.1)	< 10 (1.3)	< 10 (3.4)
Substance use disorder (%)	11 (4.1)	35 (14.3)	61 (17.0)	39 (9.4)	< 10 (5.3)	12 (13.8)
Eating disorder (%)	< 10 (2.6)	< 10 (1.6)	13 (3.6)	10 (2.4)	< 10 (3.9)	< 10 (1.1)
Attention deficit hyperactivity disorder (%)	< 10 (1.1)	10 (4.1)	11 (3.1)	10 (2.4)	< 10 (1.3)	< 10 (3.4)
Other mental disorder (%)	26 (9.6)	41 (16.8)	59 (16.4)	45 (10.8)	< 10 (11.8)	10 (11.5)
Suicide attempt (%)	18 (6.6)	21 (8.6)	33 (9.2)	41 (9.8)	< 10 (10.5)	< 10 (10.3)
Cancer (%)	11 (4.1)	< 10 (2.0)	< 10 (2.5)	13 (3.1)	< 10 (3.9)	< 10 (3.4)

Asthma(%)	< 10 (1.5)	< 10 (1.6)	< 10 (1.1)	0 (0.0)	< 10 (2.6)	< 10 (1.1)
Other respiratory disease (%)	22 (8.1)	21 (8.6)	38 (10.6)	34 (8.2)	< 10 (9.2)	< 10 (4.6)
Musculoskeletal disease (%)	30 (11.1)	27 (11.1)	43 (12.0)	46 (11.0)	< 10 (9.2)	11 (12.6)
Other somatic disorder (%)	168 (62.0)	160 (65.6)	243 (67.7)	277 (66.4)	44 (57.9)	52 (59.8)
**Country of birth (%)**						
Afghanistan	16 (5.9)	10 (4.1)	18 (5.0)	27 (6.5)	7 (9.2)	< 10 (8.0)
Africa	13 (4.8)	17 (7.0)	21 (5.8)	43 (10.3)	< 10 (10.5)	< 10 (9.2)
Iraq	59 (21.8)	66 (27.0)	73 (20.3)	105 (25.2)	17 (22.4)	23 (26.4)
Iran	21 (7.7)	21 (8.6)	39 (10.9)	39 (9.4)	< 10 (5.3)	< 10 (6.9)
Other Asian	18 (6.6)	26 (10.7)	45 (12.5)	45 (10.8)	13 (17.1)	< 10 (8.0)
Other European Countries	111 (41.0)	75 (30.7)	124 (34.5)	122 (29.3)	22 (28.9)	23 (26.4)
Rest of the world	33 (12.2)	29 (11.9)	39 (10.9)	36 (8.6)	< 10 (6.6)	13 (14.9)

[1] Big cities—Stockholm, Gothenburg, and Malmö; medium-sized cities—cities with more than 90,000 residents within 30 kilometers of the city center; small cities/villages—remaining cities or villages in Sweden.
[2] 166 individuals have missing education information
[3] Previous healthcare or treatment was dichotomized by the mean of inpatient/outpatient stay

Table S4 Mean cumulative sum of dosages in each medication by averaging the weekly defined daily doses (DDD) over a two-year interval using Louvain cluster memberships among refugee and Swedish-born young adults

	Cluster membership	N (%)	antidepressants	antipsychotics	anxiolytics	sedative-hypnotics	mood stabilizers
Refugee (1,454)	1	271 (19.3)	170	2.6	21	12	0.76
	2	244 (17.4)	280	24	16	36	4.5
	3	359 (25.6)	310	21	38	82	8.6
	4	417 (29.7)	150	5.8	14	73	2.8
	5	76 (5.4)	54	0.0	0.0	0.0	0.0
	6	87 (6.2)	200	0.0	0.096	0.0	0.0

	Cluster membership	N (%)	antidepressants	antipsychotics	anxiolytics	sedative-hypnotics	mood stabilizers
Swedish-born[1] (n=11,018)	1	2179 (20.1)	300	4.1	27	18	3.3
	2	1795 (16.6)	530	14	19	42	16
	3	3528 (32.6)	460	17	46	92	12
	4	2367 (21.9)	290	6.2	23	88	3.7
	5	343 (3.2)	73	0.0	0.12	0.058	0.0
	6	806 (7.4)	300	0.0	0.025	0.025	0.0

[1]Swedish-born indicates a native-born Swede with parents who were born in Sweden

Figure S1 Time-dependent medication use in defined daily doses (DDD), summed over each of the considered medication categories, within two-year interval centered around the diagnosis of common mental disorder (CMD), 1,454 refugees and 11,018 Swedish-born with a CMD diagnosis aged 18 to 24 from 2007 to 2013 in Sweden

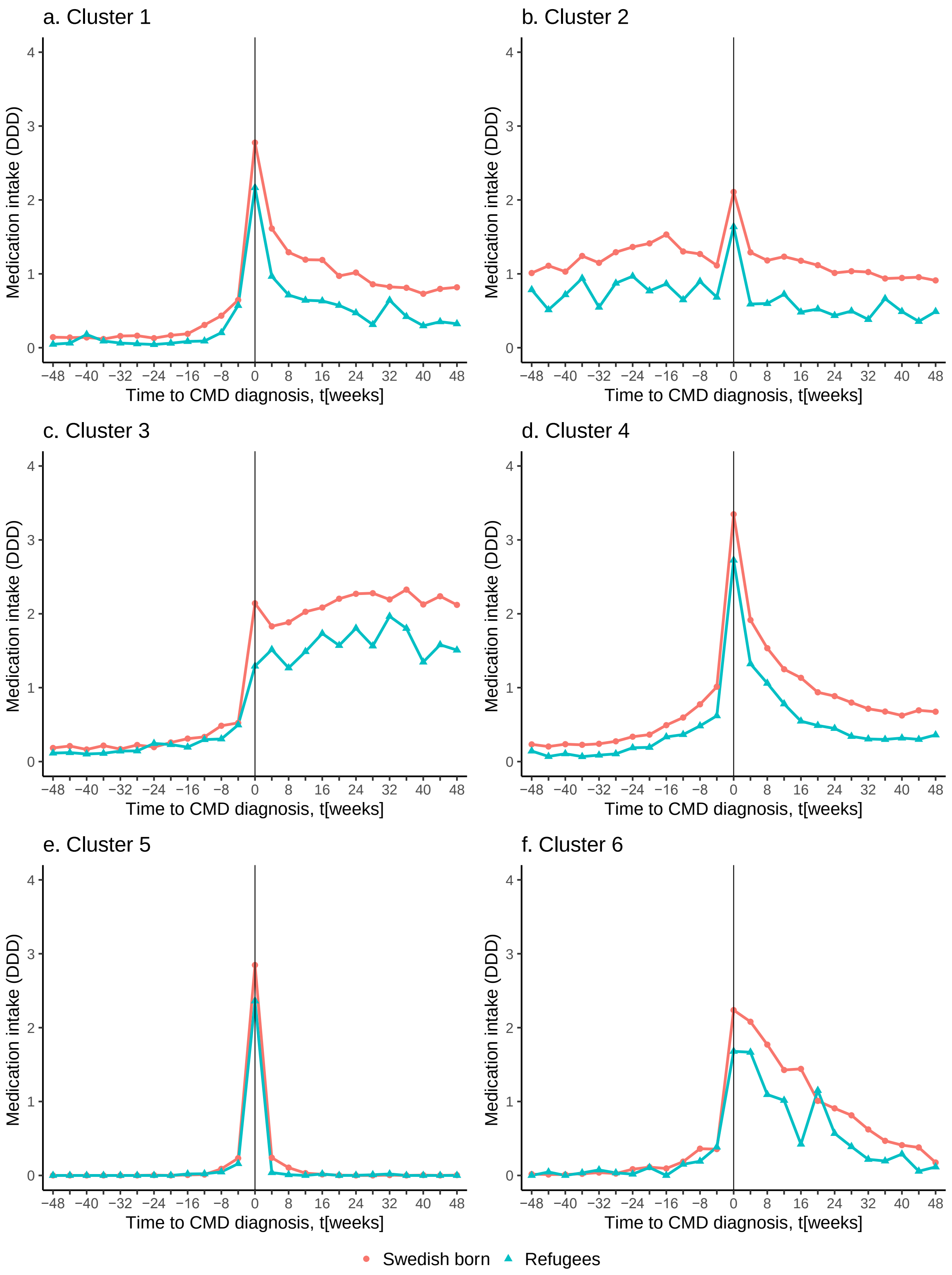

Figure S2 Time-dependent medication use in defined daily doses (DDD) summed over all medication categories within two-year interval centered around the diagnosis of common mental disorder (CMD), 7,734 individuals with anxiety disorder and 4,738 with depression at age 18 to 24 from 2007 to 2013 in Sweden

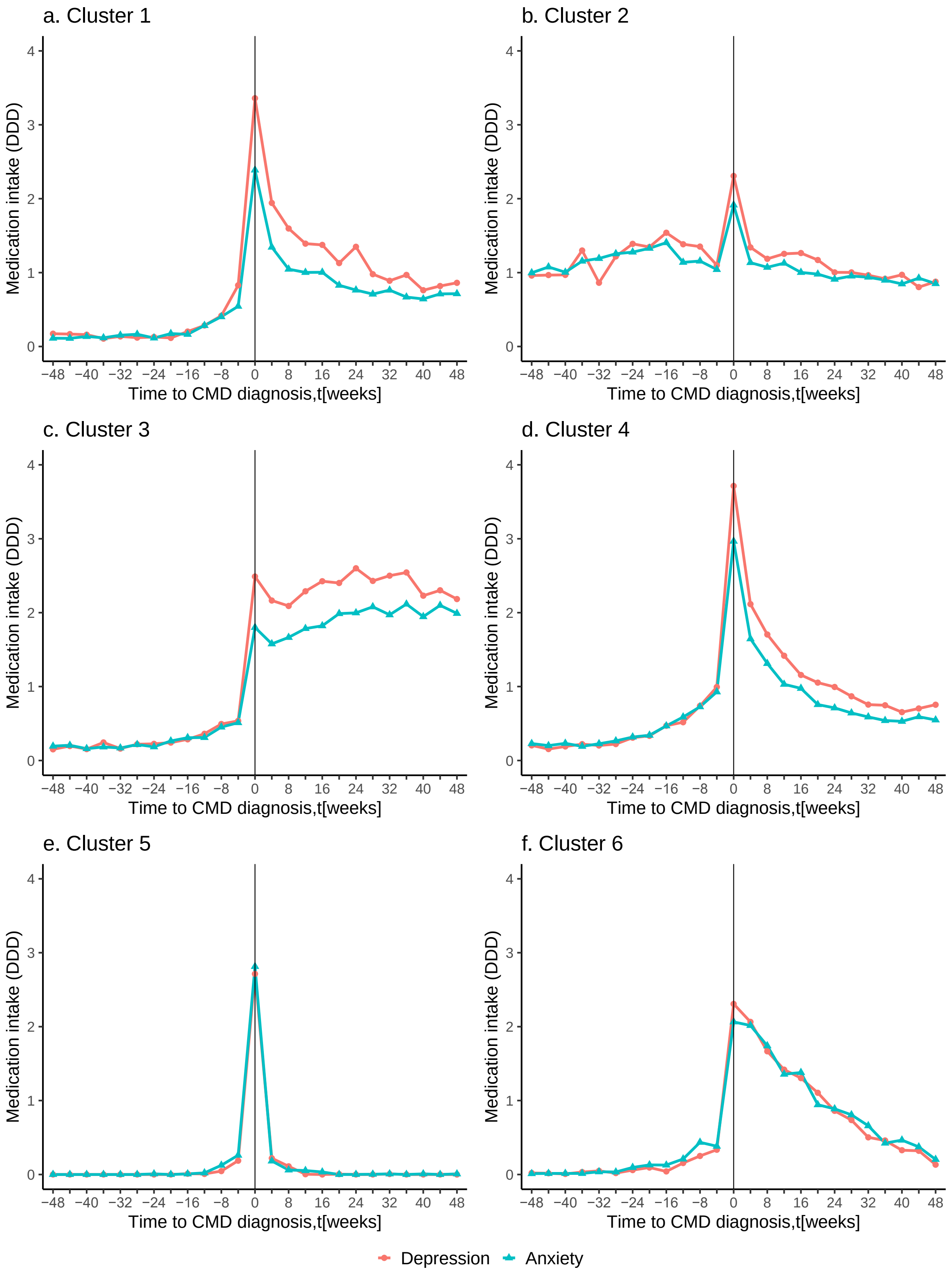

Figure S3 Time-dependent medication use in defined daily doses (DDD) for each medication category by cluster within two-year interval centered around the diagnosis of common mental disorder (CMD), 1,454 refugee youth with a CMD diagnosis at age 18 to 24 from 2007 to 2013 in Sweden

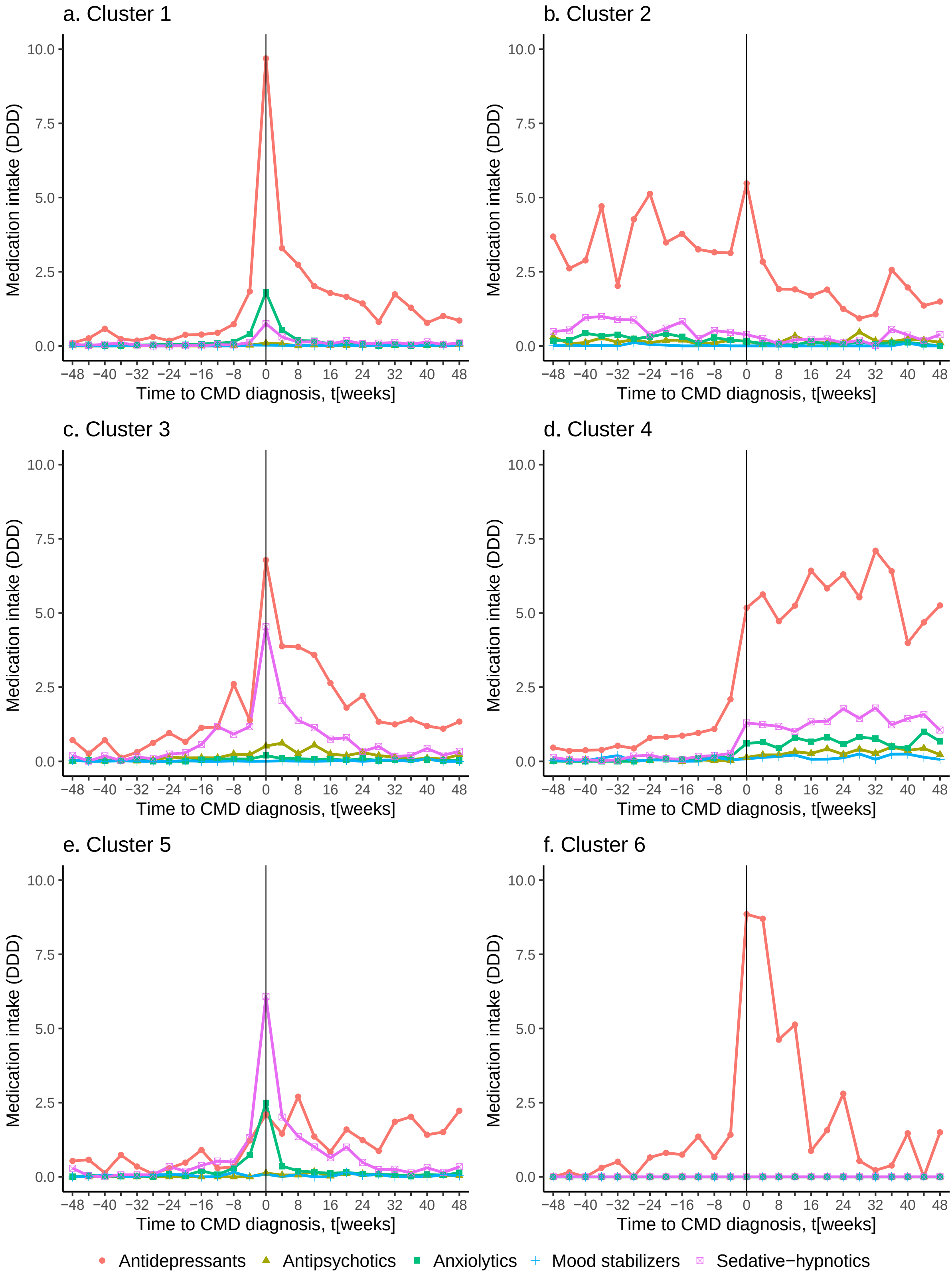

Figure S4 Time-dependent medication use in defined daily doses (DDD) for each medication category by cluster within two-year interval centered around the diagnosis of common mental disorder (CMD), 11,018 Swedish-born youth with a CMD diagnosis at age 18 to 24 from 2007 to 2013 in Sweden

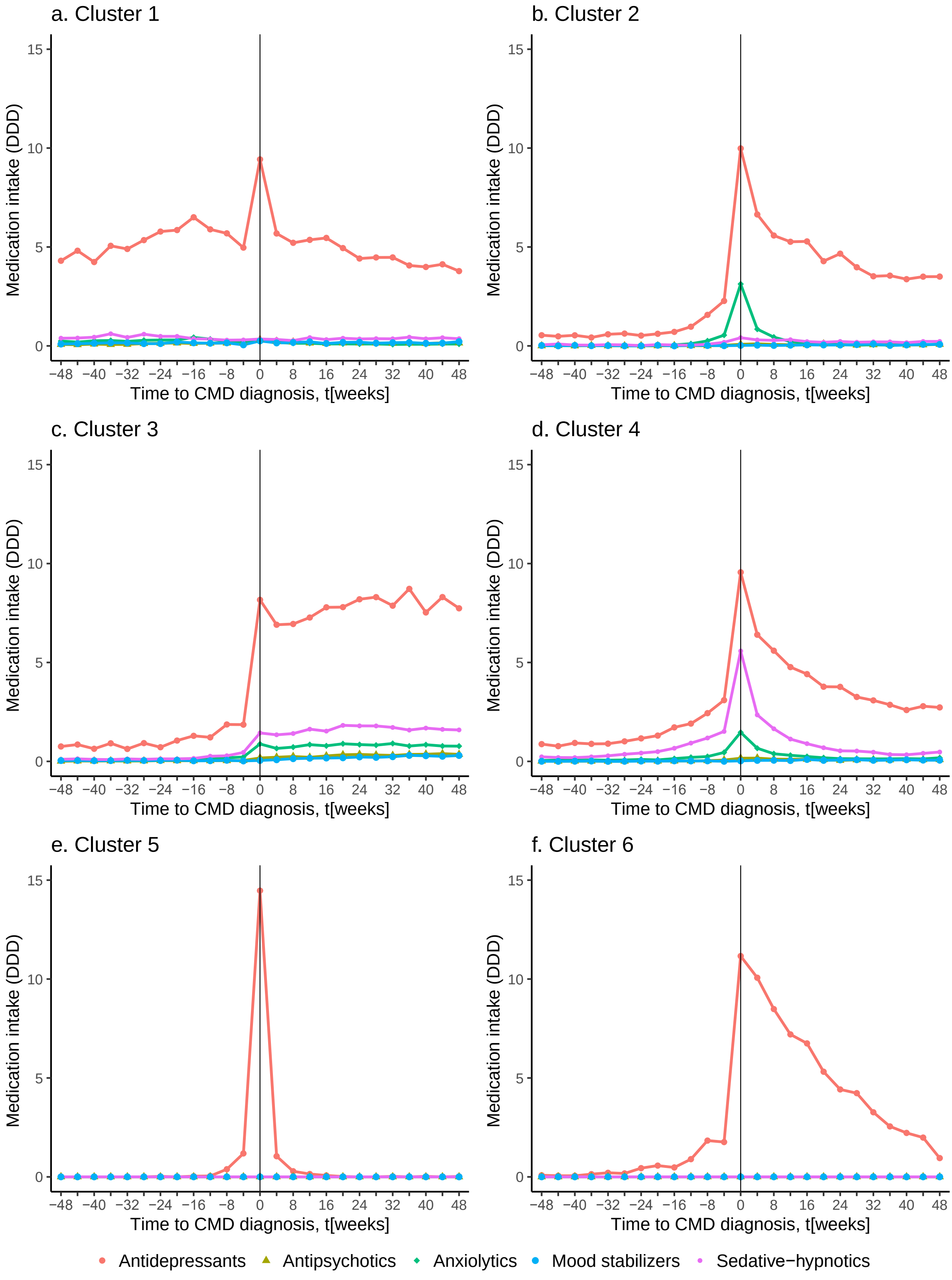

Figure S5 Time-dependent medication use in defined daily doses (DDD) for each medication category by cluster within two-year interval centered around the diagnosis of common mental disorder (CMD), 7,734 individuals with anxiety disorder at age 18 to 24 from 2007 to 2013 in Sweden

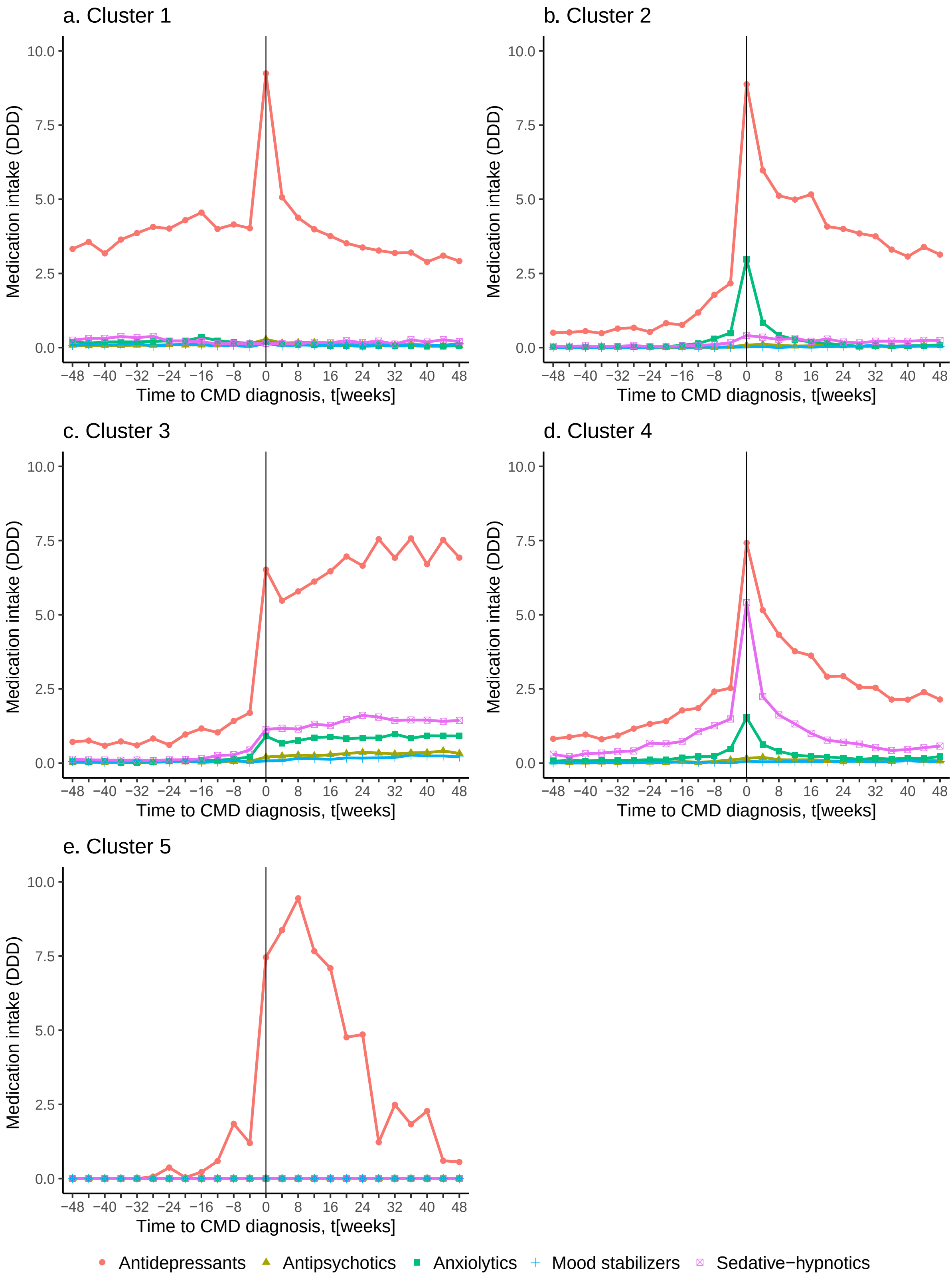

Figure S6 Time-dependent medication use in defined daily doses (DDD) for each medication category by cluster within two-year interval centered around the diagnosis of common mental disorder (CMD), 4,738 individuals with depression at age 18 to 24 from 2007 to 2013 in Sweden

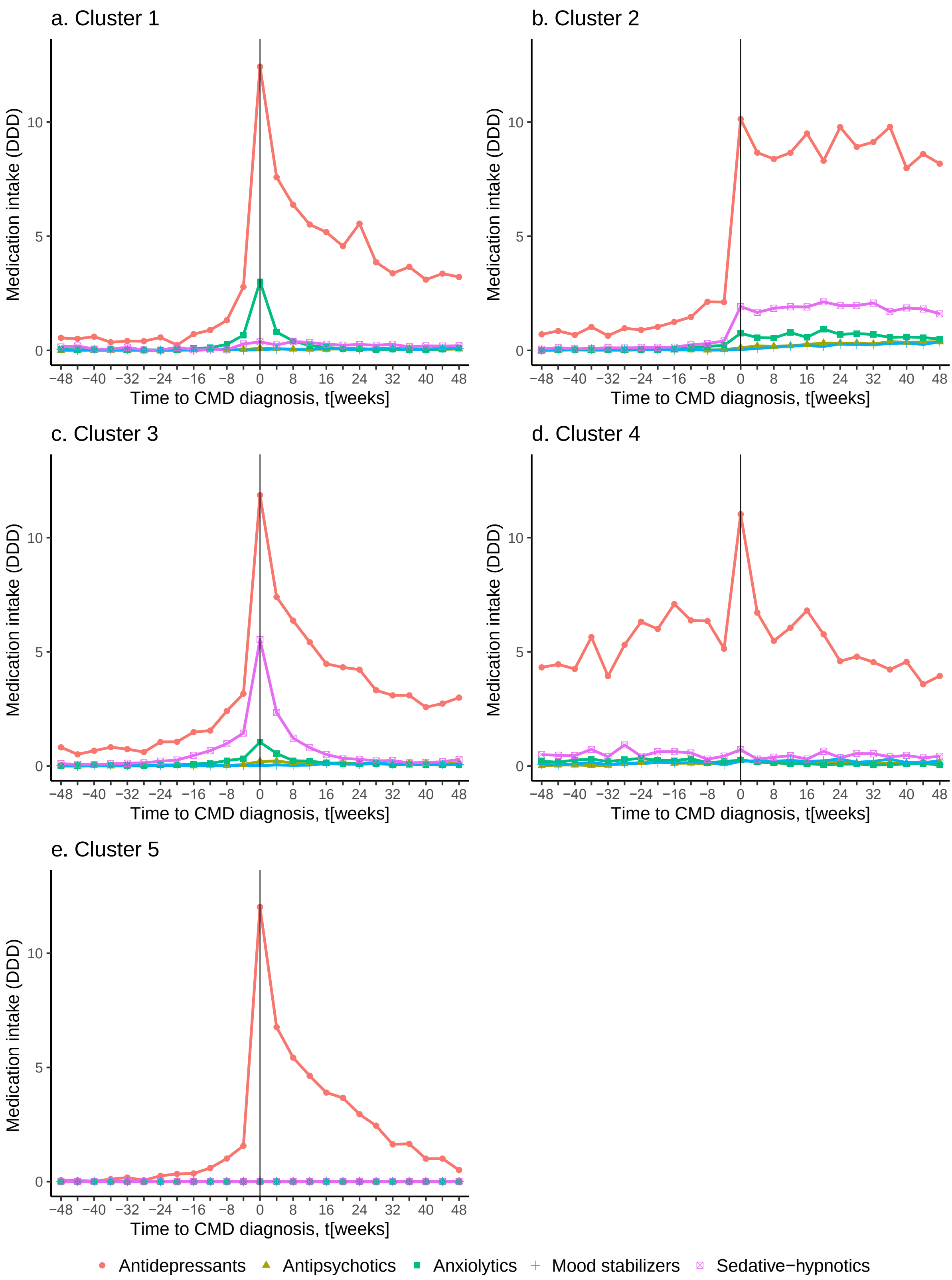

a. Cluster 1
b. Cluster 2
c. Cluster 3
d. Cluster 4
e. Cluster 5
Medication intake (DDD)
0
5
10
−48
−40
−32
−24
−16
−8
0
8
16
24
32
40
48
Time to CMD diagnosis, t[weeks]
Antidepressants
Antipsychotics
Anxiolytics
Mood stabilizers
Sedative−hypnotics
